# Colorectal Cancer Blood-Based Biomarkers

**DOI:** 10.1155/2017/2195361

**Published:** 2017-09-25

**Authors:** Nina Hauptman, Damjan Glavač

**Affiliations:** Department of Molecular Genetics, Institute of Pathology, Faculty of Medicine, University of Ljubljana, Ljubljana, Slovenia

## Abstract

Mortality and morbidity associated with colorectal cancer (CRC) are increasing globally, partly due to lack of early detection of the disease. The screening is usually performed with colonoscopy, which is invasive and unpleasant, discouraging participation in the screening. As a source of noninvasive and easily accessible biomarkers, liquid biopsies are emerging. Blood-based biomarkers have the potential as diagnostic and prognostic tool in CRC. Early stage detection of CRC with high sensitivity and specificity would likely lead to higher participation in the screening test. It would also improve the prognosis of the disease and improve the recurrence risk. In this review, we summarize the potential biomarkers for early detection and monitoring of CRC.

## 1. Introduction

Colorectal cancer (CRC) is a malignant neoplasm of the colon, rectum, and appendix and is a major health burden, causing one-third of cancer-related deaths [1]. The time of diagnosis greatly influences the overall survival rate of patients with CRC. Five-year survival rates are estimated to be between 85 and 90% for patients with localized cancer to colon or rectum. Survival decreases significantly for patients with distant metastasis, with 5-year survival of only 12.5%. Appendix cancers exhibit higher survival rates in all stages [1]. The number of cases of CRC has decreased due to advances in screening and diagnostic methods [2].

CRC develops as a result of genetic and epigenetic alterations, as well as environmental factors. CRC occurs as familial, inherited, and sporadic disease, where less than 10% reported cases are inherited CRCs. Lynch syndrome, familial adenomatous polyposis (FAP), and Peutz-Jeghers syndrome are inherited diseases which have a predisposition to progress to CRC. Familial form of CRC accounts for 25% of all cases. The largest group is the sporadic form of CRC, which accounts for 70% of all CRCs. With this form of CRC etiological, dietary and environmental factors are connected [3].

CRC is a heterogeneous disease, arising through different molecular mechanisms. Currently, three major molecular mechanisms through which CRC carcinogenesis can evolve are chromosomal instability (CIN), microsatellite instability (MSI), and the CpG island methylator phenotype (CIMP). Imbalances in chromosome number and a loss of heterozygosity (LOH) are characteristic for CIN molecular subtype [3]. Mutations in mismatch repair genes, such as *MLH1*, *MSH2*, and *MSH6*, are characteristic for MSI. MSI molecular subtype is related to inherit CRC. CIMP is found in sporadic cases of CRC and is characterized by aberrant methylation of tumor suppressor genes silencing them.

The survival of CRC affected patients depends highly on early detection [4]. The main screening approaches include direct structural examinations and fecal tests. Fecal tests are used to detect blood in the stool, which can be detected with hemoglobin test or immunohistochemistry. Fecal test is not specific for CRC and direct structural exam, such as flexible sigmoidoscopy and colonoscopy, must succeed fecal test. The structural examinations are invasive procedures and reduce the willingness of a patient to participate in CRC screening.

Improving the patients' prognosis, treatment response prediction, and recurrence risk would be enabled with reliable biomarkers for early detection of CRC. Carcinoembryonic antigen (CEA) is the most widely used biomarker for CRC in the clinical setting but has limited sensitivity and specificity [5]. In recent years, “liquid biopsies” are becoming recognized as sources of novel blood-based biomarkers. Detection of biomarkers in a patient blood sample would provide the most practical screening tool. The advantages of detecting a biomarker in blood or other bodily fluids include minimal invasiveness and easy accessibility. In this review, we summarize the currently known circulating biomarkers that may potentially be used for the early detection of CRC and monitoring the prognosis of CRC patients. These biomarkers include circulating tumor cells, DNA, RNA, and proteins [6].

## 2. Currently Available Blood-Based Tests

Extensive efforts have been made to identify blood-based markers for the early detection of CRC. Most of the candidate markers have been evaluated in clinical settings and are mostly detected in advanced stages of CRC. Noninvasive blood-based tests promise to improve screening and reduce mortality of this malignancy. The most widely used blood-based CRC biomarker is carcinoembryonic antigen (CEA), although it has proved to be a valuable tool for patient monitoring.

### 2.1. Carcinoembryonic Antigen

Blood-based CRC-specific antigens are a topic extensively researched. However, there are only two biomarkers available to monitor CRC patients, carcinoembryonic antigen (CEA) and carbohydrate antigen 19-9 (CA 19-9). CEA is a high molecular weight glycoprotein, discovered in 1965, and found in embryonic tissue and colorectal tumors. It is a biomarker to monitor CRC recurrence. CEA elevated levels are a poor prognostic factor for resectable CRC correlating to cancer progression [7]. The sensitivity of this marker increases with tumor stage [8], while levels decrease after tumor resection. High levels of CEA in blood are not specific for CRC but can also include inflammatory bowel disease, pancreatitis, liver disease, or other malignancies. Interestingly, elevated levels of CEA are detected in advanced stages of CRC in a fraction of all CRC patients; therefore, CEA levels are not an effective method of screening.

The CA19-9 antigen is less sensitive and specific for CRC compared to CEA, but it is a marker available to detect pancreaticobiliary malignancies. CEA is still the antigen of choice to use as a prognostic marker after diagnosis and to monitor disease progression.

Tissue polypeptide-specific antigen (TPS) and tissue polypeptide antigen (TPA) which detects the fragments of cytokeratins 8, 18, and 19 lack the sensitivity and specificity for CRC screening. Most of the studies report increased levels of TPA and TPS in the metastatic stage of CRC. Some studies suggested that the combination of TPA and CEA has greater sensitivity and can identify recurrence in CRC patients [9–12].

## 3. Circulating Tumor Cells

Circulating tumor cells (CTCs) are found in peripheral blood and are tumor cells that have separated from primary tumor or metastasis. CTCs are found in almost all cancers, such as colon, breast, prostate, lung, ovary, pancreas, liver, gastric, esophageal, renal, bladder, thyroid, nasopharyngeal, and melanoma. CTCs are usually not found in patients without cancer and can usually be detected before the metastasis occurs [13, 14]. Although it was discovered that up to 1 million tumor cells enter the circulation per gram of tumor tissue, most of the cells undergo apoptosis induced by cell detachment [15–21]. A small portion of cells might form micrometastasis, most of which disappear and few cells progress to form macroscopic tumors [17, 20]. The growth requires the attachment of tumor cells to the vascular endothelium, where cancer cell aggregation can occur at sites of attachment [22, 23]. The epithelial-mesenchymal transition (EMT) may also be a key step for CTCs to spread and form metastasis [24–30]. In breast cancer and prostate cancer, CTC analysis found expressed genes related to EMT; also, CTCs were commonly associated with metastatic disease [31]. Tumor cells also interact with platelets, which also can contribute to mesenchymal transition leading to metastasis [32]. Whether EMT is necessary for metastasis development is not clear, since cell invasion can occur in the absence of EMT [33, 34]. A recent report supports evidence that a small subset of CTCs is able to induce metastasis, where the cells express epithelial cell adhesion molecule (EpCAM), CD44, CD47, and MET [35]. Further research is needed to answer these questions fully.

Genomic analyses of single cells from primary and metastatic tumors are genetically diverse [36, 37]. Mutations occurring in *KRAS*, *BRAF*, and *PIK3CA* in single CTCs are heterogeneous among CTCs from the same patient. Identifying different populations of CTCs is important for therapy response [38, 39]. Furthermore, CTCs have different characteristics from those cells that were able to form a metastasis [40].

## 4. Innovative Blood-Based Tests

Patients with diagnosed advanced stage of CRC have a poor prognosis. The key to reduce morbidity and mortality associated with this disease is early detection. Availability of noninvasive, blood-based biomarkers would increase screening and tumor detection at earlier and more treatable stage. With technological advances and more comprehensive understanding of the molecular mechanism contributing to colorectal carcinogenesis, intense effort has been put into identifying CRC biomarkers detectable in blood. Circulating nucleic acids, proteins, and tumor cells have been evaluated as CRC diagnostic tools with promising levels of sensitivity and specificity. However, a new blood-based biomarker for CRC has not yet been implemented in the clinic.

### 4.1. Circulating DNA-Based Biomarkers

Cell-free DNA (cfDNA) was found in blood over 60 years ago [41]. Recently, cfDNA is opening a new field of potential biomarkers; however, high levels of cfDNA are observed also in patients with inflammatory and autoimmune diseases, besides cancer [29, 42]. Cell-free DNA is found in serum or plasma, and it originates from apoptotic and necrotic cells, although release of DNA from living cells can also occur [43].

Quantifying serum or plasma cfDNA has been shown to be significantly elevated in CRC patients. The levels decreased over the course of follow-up and disease-free patients. Increased levels of cfDNA were observed in patients with cancer recurrence or metastasis [44]. Recent studies confirmed that levels of cfDNA in CRC patients are significantly higher than those in healthy subjects. Serum levels of cfDNA also increase with tumor stage and fluctuate during chemotherapy [45, 46]. Quantification of cfDNA can be a useful tool for monitoring of CRC patients. However, circulating cfDNA amount is influenced by various issues. Amount of cfDNA was observed to be higher in serum than in plasma due to white blood cells clotting in serum. This means that plasma has a higher amount of tumor cfDNA, since extraction of cfDNA from serum might contain also wild-type DNA [47, 48]. Another thing is that circulating cfDNA is unstable, with a half-life ranging from 15 minutes to several hours [49]. Therefore, the amount of circulating cfDNA does not provide consistent results.

Other characteristics of cfDNA can be assessed to obtain more information, such as cfDNA integrity, point mutations, microsatellite alterations, and hypermethylation of gene promoters.

### 4.2. cfDNA Integrity

cfDNA is uniformly truncated when released from apoptotic cells; when released from necrotic tumorous cells, it varies in size from 185 to 200 bp long fragments [43, 50]. The integrity of cfDNA is measured as a ratio of long fragments to short fragments of cfDNA. CRC studies report different findings: some report decreased cfDNA fragmentation [51, 52], while others increased cfDNA fragmentation [53, 54]. Although reports are inconsistent, the recent study describes significantly less fragmented cfDNA in metastatic CRC compared to primary CRC [55]. This is promising information for using integrity of cfDNA as a monitoring tool for evaluation of progression of CRC.

### 4.3. Microsatellite Alterations

Microsatellites are repeated 1–6 bp units of DNA sequence in coding or noncoding regions. Microsatellite instability (MSI) is deletion or insertion of microsatellite units, which causes alterations associated with cancer. MSI in CRC is detected in 15% of all CRCs and is associated with defects in DNA mismatch repair genes. MSI is associated with hereditary nonpolyposis CRC, although most cancers with high level of MSI are sporadic [56]. Cancers with high level of MSI also show increased resistance to chemotherapeutic agents [57]. Many studies focused on detection of MSI in cfDNA [58, 59], where MSI markers were detected in cfDNA in 35% of patients with CRC; however, the detection rate varies from 0 to 60% in studies.

### 4.4. Genetic Alterations in cfDNA

Early research on point mutations of cfDNA focused on *APC* gene [60, 61]. It was shown that 8% of APC gene fragments are mutated and that detection of cfDNA was sensitive enough to identify residual disease following surgical resection [61]. Another study showed that serum detection rates of genes APC, KRAS, and TP53 were 30.4%, 34.0%, and 34.2%, respectively [62]. Usually, the mutations are unique for each patient, which prevents the development of panel covering all the somatic mutations at a low cost [63].

### 4.5. Aberrant DNA Methylation

Methylation of DNA occurs when a methyl group is covalently added to 5-carbon position on cytosine in CpG dinucleotides. Both DNA hypermethylation and hypomethylation can contribute to carcinogenesis. DNA hypermethylation usually occurs in CpG islands and refers to methylation of normally unmethylated regions. Result of hypermethylation is gene silencing, affecting DNA repair genes and tumor suppressor genes [64]. Hypomethylation is a loss of methylation compared to healthy methylation pattern and results in genomic instability [65]. Both hyper- and hypomethylation were observed in the early stages of carcinogenesis [66–68]; with advanced technologies that enable detection of genome-wide methylation, the promise of early stage methylation biomarkers has arisen [69]. The plasma and serum of CRC patients have been studied for aberrant methylation patterns for diagnostic and prognostic use. The most known is the *SEPT9* gene, encoding a guanosine triphosphate enzyme. Validation of *SEPT9* methylation biomarker has shown that the sensitivity is 90% and the specificity is 88% [70]. Currently, three commercial tests based on *SEPT9* methylation for CRC screening are available: Epi proColon 2.0 (Epigenomics), ColoVantage™ (Quest Diagnostic), and RealTime ms9 (Abbott).

Epi proColon has developed from version 1.0 to 2.0 where the new version has fewer handling steps, shorter time to result, and increased clinical performance compared to the first generation test. From a report on a recent trial, the test has detected 48.2% of cancers, with a specificity of 91.5%, and sensitivity for advanced cancers was 11.2% [71]. Further studies are needed to discover limitations of these assays and improve their sensitivity and specificity [67]. Also, other genes have been reported to be highly specific and sensitive cfDNA CRC biomarkers, such as *APC*, *CDKN2A/P16h*, *MLH1*, *ALX4*, *TMEFF2*, *NGFR*, *FRP2*, *NEUROG1*, *TPEF/HPP1*, and *RUNX3* [72–74]. Beside diagnostic, also prognostic biomarkers are needed, such as *HLTF* gene. Methylation of HLTF was reported to strongly correlate with tumor size, metastatic disease, and tumor stage; furthermore, it was associated with disease recurrence [75, 76]. Other genes with prognostic value include *HPP1* and *DFNA5* [76, 77]. Combination of these markers and further validation is needed to bring these biomarkers to clinical practice [78].

### 4.6. Circulating RNA-Based Biomarkers

In blood, RNA molecules are exposed to RNase, which rapidly degrade RNAs, making the search for RNA-based biomarkers a difficult task. Cell-free RNAs have been detected in many bodily fluids, including serum/plasma, saliva, cerebrospinal fluid, synovial fluid, tear fluid, amniotic fluid, and urine [79]. In blood, mRNA, miRNA, and lncRNA can be detected.

### 4.7. mRNA Markers

mRNA cancer biomarkers have been studied by many groups. mRNA that was analyzed was obtained from peripheral blood cells or from either serum or plasma [80]. It was demonstrated that detection of tumor mRNA is possible in the serum of malignant melanoma patients [81]. Another study employed multiplex RT-qPCR to determine the expression of CEA, cytokeratin 20, and epidermal growth factor receptor (EGFR) on RNA obtained from peripheral blood [82]. Other studies include survivin and telomerase reverse transcriptase (TERT) [83, 84]. These studies have shown that these molecules have diagnostic and prognostic value [85]. However, this research usually remained at the pilot stage. There is, however, one blood-based, ColonSentry used as a risk assessment test. The panel includes seven genes (*ANXA3*, *CLEC4D*, *LMNB1*, *PRRG4*, *TNFAIP6*, *VNN1*, and *IL2RB*) and is tested on RNA extracted from peripheral blood [86]. The genes were selected from expression profiling of 196 genes performed on 112 CRC patients and 120 controls. This panel was validated several times. The Canadian population of 202 CRC patients and 208 controls had a sensitivity of 72% and specificity of 70%. The Malaysian population of 99 CRC patients and 111 controls had a sensitivity of 61% and specificity of 77% [87]. The test was ultimately verified with 727 CRC patients of all stages, although it appears that high-risk individuals were not included in the test.

### 4.8. MicroRNAs

MicroRNAs (miRNAs) belong to the group of noncoding RNAs. They are 18–25 bp long and bind to the 3′ UTR part of mRNA with their complementary sequence, which results in translational silencing or repression of genes. They play important roles in many biological processes, such as cell proliferation, differentiation, and apoptosis [88]. Many miRNA genes are located at chromosomal regions that undergo deletions, amplifications, or translocations, making their expression patterns aberrant. Deregulated expression of miRNA was reported in many cancers, also in CRC, where they can act as tumor suppressors or oncogenes, depending on their target genes [89–91].

miRNA express higher stability in blood, compared to mRNA, due to their protection from degradation by endogenous RNase activity and resistance to extreme pH values [92]. miRNA stability is achieved due to their binding to high-density lipoproteins and their inclusion in exosomes [93]. Since their stability and evidence of their deregulation in cancers, miRNAs are promising as noninvasive biomarkers in cancer [94].

miRNA extracted from serum or plasma of CRC patients are first profiled and subsequently validated with RT-qPCR. Profiling of 742 miRNA on CRC samples and healthy controls resulted in validation of miR-601 and miR-760 on 90 CRC samples, 43 advanced adenoma (AA) samples, and 58 healthy controls (Table 1). Both miRNA had a lower expression in CRC and AA samples compared to healthy controls with a sensitivity of 83.3% and specificity of 69.1% [95]. Another research group performed profiling of 743 miRNA on CRC, AA, and healthy control samples, followed by validation with 42 CRC, 40 AA, and 53 healthy control samples. A panel of six miRNA (miR-15b, miR-18a, miR-19a, miR-19b, miR-29a, and miR-335) was successfully distinguished between CRC samples and healthy controls with the sensitivity of 78.6% and specificity of 79.3%. Furthermore, miR-18a was able to distinguish between AA samples and healthy controls with a sensitivity of 80% and specificity of 80% [96]. After profiling of 380 miRNA, another research group proposed a panel of eight miRNAs (miR-15b, miR-17, miR-142-3p, miR-195, miR-331, miR-532-5p, miR-532-3p, and miR-652), which was validated on a cohort of 45 CRC, 16 AA, and 26 healthy individuals, and found to distinguish between AA and controls with a sensitivity of 88% and specificity of 64%. The same group proposed a three-panel miRNA (miR-431, miR-15b, and miR-139-3p), which can distinguish between stage IV CRC and control samples with a sensitivity of 93% and specificity of 74% [97]. Ahmed et al. reported to validate a set of fifteen miRNA, of which nine (miR-7, miR-17-3p, miR-20a, miR-21, miR-92a, miR-183, miR-196a, and miR-214) were upregulated and six (miR-124, miR-127-3p, miR-138, miR-143, miR-146a, and miR-222) were downregulated in CRC patients' plasma and tissue with the sensitivity of 90% and specificity of 95% [98].

Some research groups have selected their candidate miRNA from literature. One study confirms miR-29a and miR-92a on 120 CRC, 37 AA, and 59 healthy individuals to differentiate between CRC and healthy individuals with a sensitivity of 83% and specificity of 84.7% [99]. Liu et al. reported miR-92a and miR-21 to be upregulated in CRC patients compared to controls with a sensitivity of 68% and specificity of 91.2%, validated on 200 CRC patients, 50 AA patients, and 80 healthy individuals [100]. Pu et al. found upregulated miR-221 in CRC with a sensitivity of 86% and specificity of 41%, validated on a cohort of 103 CRC and 37 healthy controls [101].

Studies with proposed miRNA panels are summarized in Table 1. Most of the studies focus on early stage detection in CRC, and some also include AA patients. In these studies, sensitivity ranges from 78% to 93%, and specificity from 41% to 95%. Also, some miRNAs were proposed in several panels, such as miR-15b, miR-17-3p, miR-18a, miR-20a, miR-21, miR-29a, and miR-92a. However, not all of these miRNA biomarkers were confirmed by other researchers. This could be due to patient population, using different endogenous controls, or different instrumentation. Nevertheless, these sets of miRNA could be further validated and evaluated for potential biomarkers.

### 4.9. Long Noncoding RNAs

Serum or plasma detected lncRNAs can be a potential biomarker in different types of tumors. One of the first reports on circulating lncRNA associated with a type of tumor was HULC, which expression was found to be upregulated in patients with hepatocellular carcinoma [102]. The plasma of gastric cancer patients has highly expressed lncRNA H19, compared to normal controls; its levels were observed to decrease after tumor resection [103]. For gastric cancer, a combination of UCA1/CUDR, LSINCT-5, and PTENP1 was reported to be of diagnostic value [104]. In esophageal squamous cell carcinoma, lncRNA POU3F3 expression in serum combined with plasma levels of squamous cell carcinoma antigen improves screening efficiency for early detection [105]. Only few reports were published on the expression of circulating lncRNAs as potential noninvasive diagnostic biomarker in CRC. In one, the expression levels of CRNDE-h transcript were significantly upregulated than in healthy controls, with a sensitivity of 87% and specificity of 93% for diagnosing CRC [106]. The other studied the combination of CCAT and HOTAIR. Both lncRNAs were found significantly upregulated in plasma of CRC patients, compared to normal controls. The combination proved to be of greater diagnostic value, with a sensitivity of 84.3% and specificity of 80.2%. More importantly, this combination was effective to detect CRC at an early stage (85%) [107].

## 5. Colitis-Associated Colorectal Cancer

Patients with extensive chronic ulcerative colitis (UC) have also increased risk for colorectal cancer. In these patients, surveillance is colonoscopy looking for dysplasia (precancer) or cancer. Colitis-associated cancer (CAC) is different from sporadic CRC. The cancer risk in patients with CAC depends on UC duration, severity of inflammation, and extent of disease [108, 109]. This method is cost-effective and able to reduce mortality and cancer incidence, but exposed to pathologist subjective evaluation [110, 111]. To increase clinical value, a molecular biomarker would be beneficial to the management of UC patients. UC is a disease undergoing cycles of inflammation and tissue repair, which results in oxidative stress, increasing reactive oxidative species (ROS) [112–115]. Accumulation of ROS causes damage to DNA, proteins, and lipids, initiating tumor. CAC progresses in steps, which are negative dysplasia, indefinite for dysplasia, low-grade dysplasia, high-grade dysplasia, and cancer [116]. Molecular alterations in UC are detectable prior to dysplasia, such as telomere shortening, chromosomal and microsatellite instability, aneuploidy, and loss of p53 [117–125]. Tumor suppressor gene p53 harbors an important early event in CAC, which occurs in 47%–85% [126, 127]. Another mechanism in CAC is the release of proinflammatory cytokines that are released by inflammation [128, 129]. Chronic inflammation releases cytokines which are involved in all stages of cancer, including initiation and progression of tumor, angiogenesis, and metastasis. The molecular link between inflammation and carcinogenesis is NF-*κ*B. Activated NF-*κ*B upregulates the expression of many proinflammatory molecules, such as adhesion molecules, cytokines, TNF-*α*, and IL-6, which play a critical role in tumor development and progression of colitis-associated cancer [127].

CAC molecular alterations are different from those of sporadic CRC. Alterations in APC occur early in sporadic CRC and are considered a late event in CAC progression or are not present at all. Mutations in p53 appear as early event in CAC, prior to dysplasia, while in CRC mutations in p53 are a late event. This suggests that pathways from CAC and CRC are different. Interestingly, it was observed that patients with UC have genomically abnormal nondysplastic mucosa, while having a neoplasia elsewhere in the colon [119, 130]. Furthermore, it was established that molecular alterations occurring in these UC patients are expression of proteins, genomic instability in repetitive DNA, and mitochondrial dysfunction, which are not present in dysplasia-free UC patients and provide the basis for development of biomarkers for diagnostic and therapeutic advances in CAC [119, 120, 131–133].

CAC seems to be driven largely by damage related to inflammation. Many biomarkers are being researched to facilitate the development and discriminate subtypes of patients with inflammatory bowel disease (IBD-ulcerative colitis versus Crohn's disease) and biomarkers for differentiating between UC patients who develop dysplasia and those who do not. Blood is easily accessible and is ideal for diagnostic purposes; moreover, it provides a wide variety of material (DNA, RNA, proteins, cells, etc.). Apart from enzyme-linked immunosorbent assays (ELISA), modified ELISA and targeted proteomics are emerging, facilitating analysis of low abundance proteins. Levels of p53 in the serum of UC patients were found higher when compared to normal controls. Elevated levels were found in 8/13 UC patients with CRC [134]. Although alteration in p53 is an early event in UC tumorigenesis, detection of p53 protein in serum showed moderate sensitivity, limiting its use as a neoplastic marker for high-risk patients.

### 5.1. miRNA in Peripheral Blood of UC Patients

Several studies were reported, where miRNA levels in peripheral blood of UC patients were studied. One study found that six miRNAs were significantly upregulated compared to normal controls, and those were miR-16, miR-21, miR-28-5p, miR-151-5p, miR-155, and miR-199a-5p, where miR-155 exhibited the highest expression of those six [135]. Another study found that 12 miRNAs were significantly upregulated in UC patients compared to normal controls, while miR-505 was downregulated in UC patients [136]. Attempts were also made to analyze miRNAs from different hematologic fractions, and seven miRNAs derived from platelets were found to be upregulated (miR-188-5p, miR-378, miR-422a, miR-500, miR-501-5p, miR­769-5p, and miR­874) [137]. This confirms that also miRNAs derived from platelets have the potential of becoming biomarkers in pathogenesis of UC.

## 6. Conclusion

Noninvasive screening of CRC could be achieved by utilizing blood-based biomarkers, which would enable early detection of the disease. They could also be used as a monitoring tool for recurrence or response to therapy. There are many studies that provided the data on blood-based markers useful for diagnosis and prognosis of CRC, including DNA, RNA, and protein-based biomarkers. Methylated septin 9, mutations in cell-free DNA, and several miRNAs extracted from plasma or serum show promising results towards the noninvasive tests for CRC. However, further validation of sensitivity and specificity is needed, to ensure the reproducibility of the results.

## Figures and Tables

**Table 1:** Potential blood miRNA biomarkers (adapted from [85]).

miRNA	Sensitivity	Specificity	Distinguish AA	Differentiate	Validation cohort (CRC/AA/healthy)	Reference
miR-601 ↓, miR-760 ↓	83.3%	69.1%	Yes	CRC from controls; AA from controls	90/43/58	[95]
miR-15b ↑, miR-18a ↑, miR-19a ↑, miR-19b ↑, miR-29a ↑, miR-335 ↑	78.6%	79.3%	Yes	CRC from controls; AA from controls	42/40/53	[96]
miR-15b ↑, miR-17 ↑, miR-142-3p ↑, miR-195 ↑, miR-331 ↑, miR-532-5p ↑, miR-532-3p ↑, miR-652 ↑	88%	64%		AA from controls	45/16/26	[97]
miR-431 ↑, miR-15b ↑, miR-139-3p ↓	93%	74%		Stage IV CRC from controls	45/16/26	[97]
miR-7 ↑, miR-17-3p ↑, miR-20a ↑, miR-21 ↑, miR-92a ↑, miR-183 ↑, miR-196a ↑, miR-214 ↑, miR-124 ↓, miR-127-3p ↓, miR-138 ↓, miR-143 ↓, miR-146a ↓, miR-222 ↓	90%	95%		CRC from controls		[98]
miR-29a ↑, miR-92a ↑	83%	84.7%		CRC from controls	120/37/59	[99]
miR-21 ↑, miR-92a ↑	68%	91.2%		CRC from controls	200/50/80	[100]
miR-221 ↑	86%	41%		CRC from controls	103/0/37	[101]
